# A rare case of endometrial polyp complicated with uterine inversion: A case report and clinical management

**DOI:** 10.1515/med-2022-0425

**Published:** 2022-03-17

**Authors:** Fang Li, Ying Liang, Mingyan Luo, Yufen Cheng

**Affiliations:** Gynecology Department, Jiangxi Maternal and Child Health Hospital, Nanchang, Jiangxi Province, China; Department of Gynecology, Jiangxi University of Traditional Chinese Medicine, Nanchang, Jiangxi Province, China

**Keywords:** uterine inversion, puberty, endometrial polyp, laparoscopic surgery, uterine-sparing

## Abstract

Uterine inversion is a rare disease that is particularly uncommon among non-puerperal women. Herein we reported the only case of uterine inversion known to us that was caused by the endometrial thickening and changes in the polypoid lesion in early puberty. The patient was admitted to our hospital because of massive vaginal bleeding, abdominal pain, and a protruding mass from the vagina. We obtained the patient history by collecting the results of various examinations (including magnetic resonance imaging and color Doppler ultrasound), accurate diagnosis was performed, and a reasonable treatment protocol was developed. She was subjected to laparoscopic uterine-sparing surgery to preserve her fertility. Uterine inversion is a rare disease, and early diagnosis and selection of appropriate treatment options are essential for patients with fertility requirements.

## 1 Introduction

Uterine inversion is a clinically rare disease in which the uterine fundus protrudes into the uterine cavity, and the endometrium is inverted from the cervical orifice into the vagina. It can be divided into puerperal uterine inversion and non-puerperal uterine inversion according to the time of occurrence [1]. The former usually occurs during the third stage of delivery due to the improper treatment of placental delivery, the loose uterine orifice, and the external forces pulling the placenta that cause the uterine fundus to turn inside out and pass through the cervical canal until it falls off into the vagina [2]. According to a recent 10-year study in the United States, there were only 2,427 cases among 8,294,279 deliveries [3]. The non-puerperal uterine has a lower incidence and often occurs in those with a reproductive history and a presence of large submucous myomas or sarcomas in the uterine cavity when the uterine orifice becomes loose during gradual delivery, and the uterine fundus is dragged and gradually inverted by uterine cavity tissues. This typically includes four stages: (1) the uterus is incomplete, the uterine fundus is inverted, and the uterine cavity is located at the uterine fundus; (2) the uterine fundus is inverted through the vulvar orifice; (3) the uterus is completely inverted with the uterine fundus protruding beyond the vaginal orifice; and (4) the uterus is completely inverted and is prolapsing out of the vulva through the vagina [4].

The most common clinical symptoms include irregular vaginal bleeding, pelvic pain, increased vaginal secretion, intermittent acute urinary retention, and even infection complicated with shock. There are three types of uterine inversion according to the extent of inversion: (1) incomplete uterine inversion (the uterine fundus collapses downward and might approach or exceed the cervical orifice, but it is still partially within the uterine cavity); (2) complete uterine inversion (the uterine fundus protrudes through the cervix and into the vagina); (3) uterine inversion and prolapse (the inverted uterine body prolapses outside the vaginal orifice). Uterine inversion can also be divided into the acute and chronic types according to the onset of the disease. Patients with acute uterine inversion have severe abdominal pain. They often exhibit shock symptoms 3–4 h after the onset of the disease, and their condition may even become life-threatening. In contrast, chronic uterine inversion is more common in patients who seek medical advice after surviving the untimely detection of acute uterine inversion.

Surgery is an important means of treatment in uterine inversion, five surgical methods are currently defined for the hysterectomy of endropion, namely, Spinelli, Kushner, Huntington, Tjalma, and Haultain [5].

Herein we reported a case of a 11–year-old patient in early puberty, without childbearing history. Uterine inversion was caused by the endometrial thickening and changes in the polypoid lesion in early puberty. No similar report has been found in the previous literature.

During the whole treatment process, we objectively collected the main complaint symptoms, various examination results, changes in the condition during the treatment process, collected information to protect the privacy of patients, tried to protect the fertility and body least affected, and the publication of the disease case solicited the opinions of the patient and their guardians.

## 2 Case report

**Figure 1:**
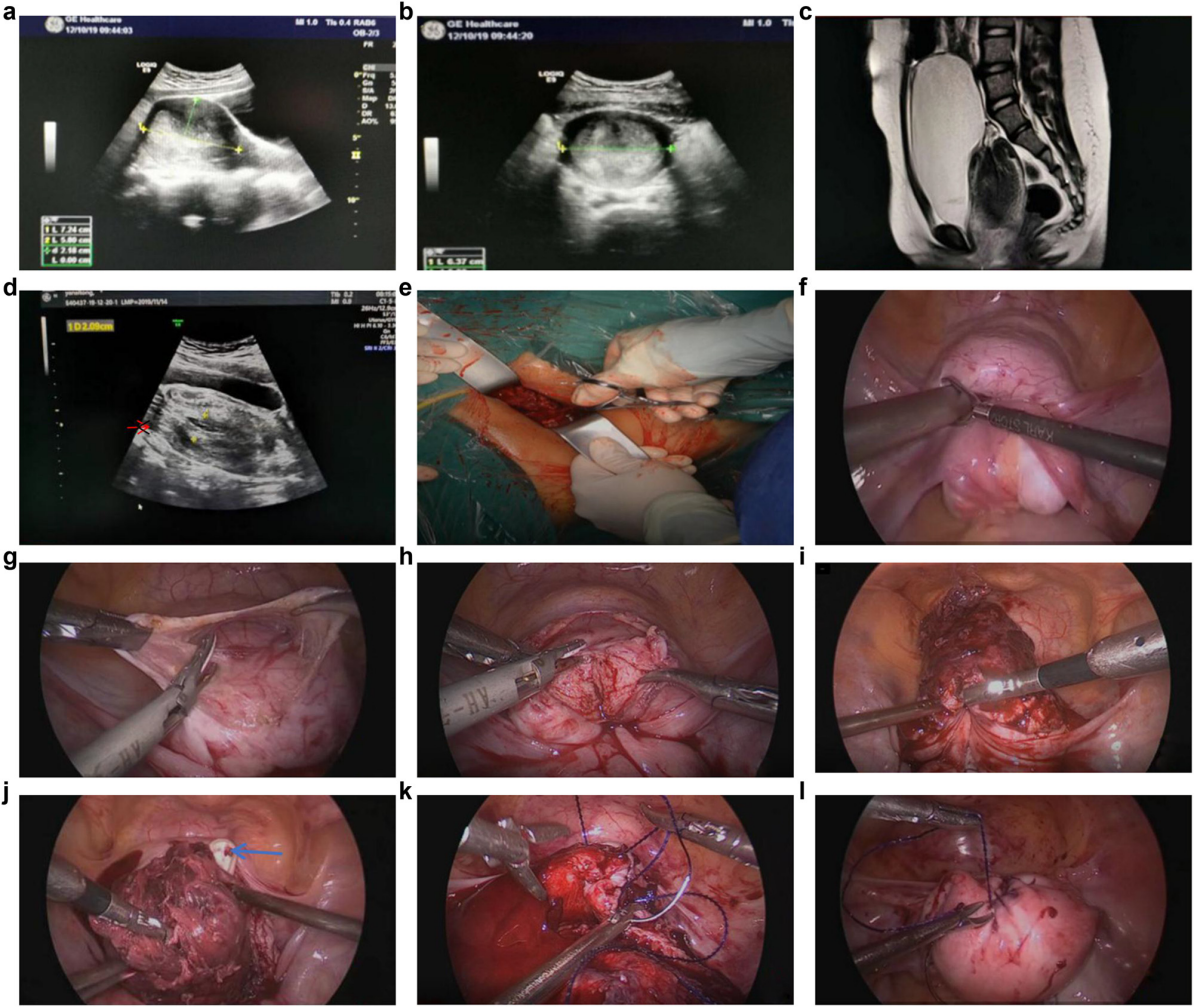
(a and b) Color ultrasound images (sagittal and transverse position) 10 days before the surgery. (c) MRI image after admission. (d) Color ultrasound image after admission (the arrow points to the ovary). (e) Intraoperative vaginal view. (f) Intraoperative laparoscopic view. (g) The uterine serosa was incised. (h) The anterior myometrium and the vagina were incised. (i and j) Uterus after incision (the blue arrow indicates the finger of the vaginal operator). (k) Suturing the vagina and the myometrium. (l) Uterus at the end of suturing.

The patient was a 11-year-old Han Chinese girl without infectious disease history or special hereditary disease history in the family. She received GNRH treatment from Jiangxi Provincial Children’s Hospital 1 year ago after being diagnosed with precocious puberty. It was reported that the color ultrasound examination at that time revealed no abnormalities (no report was seen). On November 4, 2019, the patient presented to the outpatient department of our hospital because of repeated menstrual disorders. The color ultrasound results on the same day showed that the uterine size was about 64 mm × 56 mm × 49 mm, and the endometrial thickness was about 23 mm. The patient was prescribed with 1 Dydrogesterone tablet, twice per day for 20 days. Vaginal bleeding decreased slightly but did not stop. On November 24, the patient came to our hospital for further consultation, and the routine blood tests showed that Hgb was 95 g/L, the color ultrasound showed that the endometrial thickness was about 35 mm, and the remaining items were approximately the same as the previous one. This time, the patient was prescribed with 1 tablet of drospirenone and ethinylestradiol tablet (II), once every 12 h. Bleeding was significantly reduced but did not stop, and the routine blood test showed that HG was 103 g/L. On December 10, the patient came to our hospital for another re-diagnosis. The color ultrasound showed (Figure 1a and b) that the uterine size was about 72 mm × 65 mm × 58 mm, with even echoes from the muscular layer, and the endometrial thickness was about 42 mm, with uneven echoes, and a dark sieve-pore liquid was visible inside, which suggested that uterus was getting bigger and the endometrium was thickening. The patient felt occasional distending pain in the lower abdomen.

On December 18, she experienced a sudden increase in vaginal bleeding, accompanied by severe pain in the lower abdomen, nausea, and vomiting, and a fleshy tissue about 6 cm in size could be seen at the vaginal orifice. On the same day, the patient came to our hospital after her family attempted to remove the fleshy tissue at home. The physical examination after admission showed a mass prolapsed from the vaginal orifice, about 6 cm in size, with a hard texture and uneven surface, and it was not possible to identify the cervical part with the vaginal palpation. The routine blood test at admission on December 18 showed that WBC was 24.57 × 10^9^/L, N was 91.20%, and Hgb was 77 g/L; the admission diagnosis was: (1) vaginal neoplasm: submucous myomas delivery? uterine sarcoma delivery? (2) Moderate anemia. The patient exhibited symptoms of urinary retention after admission, which were spontaneously relieved afterward, abdominal pain was gradually relieved, and the protruded mass returned to the vagina. On the day of admission, a partial tissue of about 3 cm × 1 cm × 1 cm in size was removed with tissue forceps and sent for pathological examination. The results showed a smooth muscle tissue with intravascular hyperplasia, dilatation, and hyperemia visible inside, complicated with inflammatory cell infiltration, and a few endometrial glands and stroma on the surface. Then, Magnetic Resonance Imaging (MRI) was performed, and the results showed (Figure 1c): (1) complete uterine inversion, which oppressed the urethra and caused urinary retention of the bladder; and (2) vaginal effusion, given the patient had moderate anemia at admission and continued to bleed after admission. A 1.5 Iu isotype red blood cell suspension was administered to correct the anemia. Since the risk of infection was increased because the tissue once prolapsed out of the vaginal orifice and the hemogram had increased, piperacillin sodium, sulbactam sodium, and metronidazole were administered through intravenous drip for anti-infectious treatment.

A color ultrasound re-examination on December 20 showed (Figure 1d) that the uterus lost its normal form, the demarcation between the uterine body and the cervix was unclear, the size was about 88 mm × 60 mm × 55 mm, the uterine fundus was inverted to the cervical external orifice, chaotic hyper-level echoes with a range of about 66 mm × 21 mm could be detected inside, striped blood flows could be seen on Color Doppler Flow Imaging, and the echoes of the two ovaries were detected in front of the internal cervical orifice. These results suggested uterine inversion.

On December 20, laparoscopic exploration was performed to get a definite diagnosis and restore the normal anatomical position of the uterus. During the surgery (Figure 1e–l), it could be seen that the uterine fundus was completely depressed for approximately 6 cm, and bilateral attachments were cohesive in the depression of the uterine fundus. Our attempt to reposition the uterus failed, so we incised the serosal layer of the anterior uterine wall and pushed down the bladder, after which we incised the entire layer of the anterior wall muscle layer from the everted bottom to about 2 cm of the vaginal segment. The intrauterine tissues were in disorder, in a status of thickening, edema, and necrosis, without clear tumor-like tissues. We removed the messy intrauterine tissues from the uterine cavity (rapid intraoperative histopathology did not indicate malignancy), repositioned the uterus, restored its anatomical position, and sutured the anterior wall myometrium and the vaginal segment continuously according to the anatomical structure of the muscular and the serosal layers. The surgery went smoothly. During the entire operation, bleeding was about 50 mL, and the uterus was beautifully stitched, The pathological examination showed that the tissue had a polypoid structure, the glands were in the shape of small round tubes or fissures, stromal cells showed mild decidual degeneration, and the internal blood vessels were significantly increased, congestive, expanded, and bleeding, thus suggesting an asynchronous reaction between endometrial glands and stroma. Antibiotics continued to be administered postoperatively for anti-infection purposes, and the patient recovered moderately well and was discharged from the hospital after 3 days. Regular follow-ups were carried out via telephone or on an outpatient basis.

A color ultrasound re-examination that was performed on January 6, 2020 showed no abnormalities with the uterine accessories (the uterus size was about 60 mm × 45 mm × 38 mm and the endometrium thickness was 5 mm). Consequently, a color ultrasound re-examination at the outpatient department of our hospital performed on March 25 showed that the uterus size was about 54 mm × 45 mm × 38 mm, and the endometrium thickness was 9 mm. No special treatment was given.

So far, the patient has been followed up for 6 months after the surgery. Her menstrual cycle lasted around 40 days with normal menstrual blood volume.


**Ethics statement:** The patient and her guardians provided consent to share this case in the present manuscript.

## 3 Discussion

Uterine inversion usually occurs in the third stage of the puerperium and has a fatality rate of approximately 15–43%. One of the common causes of non-puerperal uterine inversion includes intrauterine neoplasm, such as uterine submucosal myoma, uterine sarcoma, and endometrial carcinoma, which are regarded as a foreign body and are gradually extruded out of the uterine cavity. Then, the myometrial tissue at the uterine fundus to which the neoplasm is attached is pulled through the loose uterine orifice and gets stuck at the uterine orifice, unable to return to the uterus. Such patients often have a pregnancy-related disease history and a relatively loose cervix. Gomez-Lobo et al. [6] reported 150 cases of nulliparae with uterine inversion from 1887 to 2006, and suggested that nulliparous uterine inversion was mainly associated with benign myoma. A 2018 systematic review of 170 case reports found that benign leiomyomas were the leading cause of chronic uterine inversion (57.2%) followed by leiomyosarcomas at 13.5%. There was no evidence of abnormalities in 9.9% of all cases [7,8]. Myoma is the most common tumor in the benign group. The most common tumors in the malignant group are sarcoma, endometrial carcinoma, and malignant mixed Müllerian tumor. In previous case reports, the youngest patient was 15 years old [6], and an immature teratoma induced the disease in the uterus. In the present study, the uterine inversion was found in a 11-year-old girl in her early puberty. The intrauterine neoplasm was not too big and was without obvious tumor tissues. Thickening and edema of intimal tissues and polypoid tissues were mainly found, and the weighted intimal tissues dragged the fundus uteri muscular layer through the cervical canal. Further observations are necessary to establish whether the patient is complicated with congenital or acquired uterine local myometrium weakness, even though direct visual inspection revealed no obvious abnormalities, and whether there is any risk factor for uterine inversion recurrence.

Uterine inversion is a rare condition that can be easily misdiagnosed as a cervical malignant tumor or uterine submucous myomas delivery, and MRI can be helpful for the diagnosis. In this case, before the admission of the patient, repeated color ultrasounds revealed no signs of uterine inversion. The results only showed uterus enlargement, endometrial thickening, and sieve-pore changes. The patient was preliminarily diagnosed with submucous myomas delivery or uterine sarcoma delivery at the sight of the protruded mass from the vagina, while the frontal plane of MRI showed a V-shaped uterine cavity and a T2-weighted MRI image showed an inverted uterus in the shape of “bulls-eye” [9,10]. A color ultrasound re-examination revealed the inverted uterus, bilateral ovaries that were cohesive at the uterine fundus, and the shrunken uterine serosal layer, which might be misjudged as thickened endometrium. It was impossible to diagnose incomplete uterine inversion with just the single-use of color ultrasound. Partial inversion of the uterus caused ischemic necrosis of local tissues and increased edema, and the use of hormone promoted cervical dilatation and the relaxation of the cardinal uterine ligament to some extent, which finally resulted in the complete inversion of the uterus. Puerperal uterine inversion is mostly found in acute cases. The uterus of the patient in the case was completely inverted and protruding beyond the vaginal orifice, reaching stage IV. In the early stage, it was not easy to diagnose the condition since patients’ abdominal pain symptoms were not obvious, and there is only vaginal bleeding. Also, this condition can be easily overlooked considering the rarity of such cases, as well as lack of effective detection means. Future studies should focus on improving the early diagnosis rate of uterine inversion, timely detection of the disease and interventions, treatment of the factors leading to further occurrence, and terminating the inversion process. The present case can be used as a reference for the diagnosis capacities of color ultrasound.

Patients with uterine inversion often suffer from severe abdominal pain, possibly accompanied by nausea and vomiting. The contraction of the cervical ring and the insufficient blood supply to the uterine pedicle lead to ischemia and necrosis of the muscle layer and tumor body, thus further increasing vaginal bleeding and the possibility of infection. Symptoms of shock that were out of proportion with hemorrhage have been frequently reported in previous cases. One possible cause is that the extensive ligament and peritoneal nerve relaxation cause an increase in vagus nerve tension, thus exerting further pressure on the ovaries when pulled into the uterine cavity. Rapid identification and proper treatment can restrain the development of the disease. In this case, the patient was anemic due to repeated bleeding, and she exhibited symptoms of obvious abdominal pain and vaginal bleeding before admission; after admission, the patient underwent immediate blood transfusions and anti-inflammatory treatment, which helped in a timely control of the patient’s disease [11]. Since the inversion of the uterus causes abnormalities in the anatomical structure of surrounding tissues, it may result in urethral obstruction and urinary retention in the patient. The treatment of uterine inversion depends on the preoperative diagnosis, patients’ fertility requirements, and the severity of the disease. Most patients with puerperal uterine inversion, have a loose cervical orifice, and some patients can be treated by repositioning the uterus through the vagina under anesthesia, and transabdominal cervical cerclage or intrauterine tamponade balloon can be performed to prevent the recurrence of uterine inversion [12,13]. For patients in whom transvaginal repositioning has failed, laparotomy is often performed to incise the anterior wall of the uterus, reposition the uterus, and restore the anatomical structure of the uterus. In their study, Della Corte et al. [14] reviewed the literature and summarized 22 non-puerperal uterine inversion cases, reporting that most of the authors used the approach of combining vaginal and abdominal surgery. The first step was to identify the causes for inversion, after which the uterus was partially or completely excised, and only 2 patients, aged 15 and 19 years, retained their uteri; the causes of their valgus were associated with uterine teratomas and submucosal fibroids, respectively. There were two factors to consider, i.e., the severity of the disease and whether the patient had any fertility requirements. There are currently five defined surgical methods for the hysterectomy of uterine inversion [5]. Surgery can be performed by opening the anterior wall (Spinelli) and the posterior wall (Kushner) through the vagina. Huntington’s method and Tjalma’s method are abdominal surgeries without incision of the uterus. In Huntington’s method, Alice forceps are used to pull the inverted part out of the uterus, while Tjalma’s method requires retroperitoneal surgery. The best option might be not to cut apart the uterus to reduce the spread of tumor lesions from an oncological perspective; yet, some of the methods are not feasible (vaginal surgery), some generally do not work (Huntington), some require retroperitoneal surgery (Tjalma). In Haultain’s method, the anatomical position of the uterus is restored by incising the cervical ring vertically at the posterior part of the cervix and slowly pulling the uterine fundus. In the present case, due to the patient’s young age, we attempted to reposition the uterus by hand laparoscopically through the vagina under general anesthesia and performed the Huntington surgery; nonetheless, we failed due to the cervical ring being too narrow. Finally, we incised the entire anterior uterine wall and the upper vaginal section laparoscopically and repositioned the uterus, and the total length of the uterine and vaginal incision was about 8 cm. During the surgery, the intrauterine neoplasm was also excised, taken out through the vagina, placed in a specimen bag, and sent for a frozen pathological examination to exclude the possibility of malignancy. It is necessary to distinguish the anatomical hierarchy of the myometrium and the vaginal wall during the surgery. If necessary, cervical cerclage can be performed to ensure the uterine will not be inverted again. To the best of our knowledge, this is the first such case in which laparoscopic uterine-sparing surgery was performed on such a young patient with uterine inversion to preserve her fertility.

While uterine inversion is a rare condition, the non-puerperal uterine inversion is even less common, which makes it difficult to be diagnosed and to give reasonable treatment at the time of clinical occurrence. Clinicians and imaging doctors should constantly sum up experience to make early diagnoses and early intervention and to ultimately stop the occurrence of inversion.

## Abbreviations

MRI: magnetic resonance imaging
